# Multi-omics analysis reveals host-microbe interactions driving divergent energy allocation strategies in Tibetan sheep under cold-season feeding regimes

**DOI:** 10.1186/s40104-025-01259-w

**Published:** 2025-09-02

**Authors:** Xungang Wang, Qian Zhang, Tongqing Guo, Shanshan Li, Yuna Jia, Shixiao Xu

**Affiliations:** 1 Key Laboratory of Adaptation and Evolution of Plateau Biota, Northwest Institute of Plateau Biology, Chinese Academy of Sciences, Xining, 810008 China; 2 University of Chinese Academy of Sciences, Beijing, China

**Keywords:** Adaptation strategy, Feeding regimes, Multi-omics, Rumen, Tibetan sheep

## Abstract

**Background:**

As an indigenous livestock species on the Tibetan Plateau, Tibetan sheep exhibit remarkable adaptability to low temperatures and nutrient-scarce environments. During the cold season, Tibetan sheep are typically managed under two feeding regimes: barn feeding (BF) and traditional grazing (TG). However, the molecular mechanisms underlying their adaptation to these distinct management strategies remain unclear. This study aimed to investigate the adaptive strategies of rumen function in Tibetan sheep to cold-season feeding regimes by integrating analyses of rumen morphology, microbiome, metabolome, and transcriptome. Twelve healthy Tibetan sheep with similar body weights were assigned into two groups (BF vs. TG). At the end of the experiment, rumen tissues were subjected to histological observation. Multi-omics techniques were employed to evaluate the effects of cold-season feeding regimes on rumen function in Tibetan sheep.

**Results:**

The ruminal papilla height, width, and muscular thickness were significantly higher in BF group. The relative abundances of Actinobacteria and *Succiniclasticum* were significantly elevated in the rumen of BF group, whereas Rikenellaceae, Gracilibacteria, and Lachnospiraceae showed higher abundances in the TG group. Metabolomic analysis identified 19 differential metabolites between the two groups, including upregulated compounds in BF group (fumaric acid, maltose, L-phenylalanine, and L-alanine) and TG group (e.g., phenylacetic acid, salicyluric acid and ferulic acid). These metabolites were predominantly enriched in phenylalanine metabolism, alanine, aspartate and glutamate metabolism, and phenylalanine, tyrosine and tryptophan biosynthesis pathways. Additionally, 210 differentially expressed genes (DEGs) were identified in rumen epithelium: 100 upregulated DEGs in the BF group were enriched in nutrient metabolism-related pathways (e.g., fatty acid degradation and PPAR signaling pathway), while 110 upregulated DEGs in the TG group were associated with immune-related pathways (e.g., p53 signaling pathway and glutathione metabolism).

**Conclusions:**

Among these, we observed distinct rumen functional responses to different cold-season feeding regimes in Tibetan sheep and revealed energy allocation strategies mediated by host-microbe interactions. In the BF group, Tibetan sheep adopted a "metabolic efficiency-priority" strategy, driving rumen microbiota to maximize energy capture from high-nutrient diets to support host growth. In contrast, the TG group exhibited an "environmental adaptation-priority" strategy, where rumen microbiota prioritized cellulose degradation and anti-inflammatory functions, reallocating energy toward homeostasis maintenance at the expense of rumen development and growth performance.

**Supplementary Information:**

The online version contains supplementary material available at 10.1186/s40104-025-01259-w.

## Background

The Tibetan Plateau, characterized by its extreme climatic conditions and unique geomorphology, represents a globally significant repository of alpine biodiversity and genetic resources. As one of Earth's most ecologically fragile yet species-rich high-altitude ecosystems, it plays a pivotal role in global biodiversity conservation [1]. Within this complex ecological matrix, grassland ecosystems dominate over 60% of the plateau's surface area, forming the principal habitat for indigenous herbivores [2]. These alpine meadows, existing under hypoxic conditions at average elevations exceeding 4,500 m, sustain a diverse assemblage of endemic ungulates including Tibetan sheep (*Ovis aries*), domestic yak (*Bos grunniens*), and wild species such as Tibetan antelope (*Pantholops hodgsonii*), and Przewalski's gazelle (*Procapra przewalskii*) [3–6]. Notably, these herbivores rely exclusively on natural grassland vegetation as their primary nutritional source. However, systematic investigations reveal significant seasonal fluctuations in forage availability and nutrient composition, particularly during the winter months when herbivores encounter dual stresses, namely low temperature and nutrient deficiency [7, 8]. Remarkably, indigenous herbivores have evolved sophisticated physiological and behavioral adaptations to mitigate these stressors through coordinated host-microbe interactions.

As an archetypal plateau-adapted ruminant, Tibetan sheep represent one of the most ancient domesticated ungulates endemic to the Tibetan Plateau, primarily inhabiting alpine pastoral zones at elevations of 3,500–5,000 m. This species has evolved specialized morphological adaptations and metabolic resilience to withstand extreme environmental stressors characteristic of high-altitude ecosystems [4, 7, 9]. During extended cold seasons (October–April), the two primary regimes employed in the rearing of Tibetan sheep are barn feeding and traditional grazing. Under conditions of barn feeding, adequate nutrient supply is ensured for Tibetan sheep. Conversely, traditional grazing poses dual challenges of low temperature and low nutrition [10]. Therefore, the study of the differences in rumen function of Tibetan sheep under different feeding regimes is of paramount importance, with the objective of optimising feeding management strategies and improving production performance.

The rumen, a pivotal digestive organ in ruminants, plays a pivotal role in host nutrient metabolism, immune regulation and health [11]. It is inhabited by a complex microbial community, comprising bacteria, protozoa, fungi, and archaea, which act synergistically to help ruminants convert indigestible plant fibres into nutrients available to the host [12]. Several researchers have demonstrated a close correlation between the structure and function of rumen microbial communities and the efficiency of nutrient metabolism, growth performance and health status of the host [13–15]. In the plateau environment, the rumen microbial community of Tibetan sheep exhibits a unique composition and function, significantly different from that of livestock at lower altitudes [16]. Specifically, the rumen of Tibetan sheep contains microorganisms that are able to efficiently degrade cellulose and hemicellulose, thereby enhancing the host's ability to utilise low-quality feeds. The advent of molecular biology and bioinformatics has led to the development of sophisticated analytical tools, such as genomics technology, which has significantly enhanced our understanding of the complex microbial ecosystem within the rumen, encompassing its structure, function, and the intricate interactions with the host [17–19]. It has been determined that host genetics exerts a substantial influence on the rumen microbiome of Hu sheep, and the collaborative host genetics-rumen microbial regulation of sheep body weight phenotype can be elucidated from the perspective of 'host genome + microbiome' [20]. A combined rumen metagenome and host transcriptomic study on the early rumen development in neonatal ruminants is already conducted, which demonstrated a significant correlation between *Prevotella*, *Streptococcus*, and *Clostridium* in the rumen and the host epithelial morphogenesis, protein localisation and protein translocation processes [21]. Consequently, it is insufficient to investigate the adaptive mechanisms of physiological metabolism in response to external environmental stresses solely from the host or microorganisms. The integration of omics technology is a necessary and effective method to analyse the adaptive physiological metabolism and molecular mechanisms of animals.

In the present study, we employed a multi-omics approach to integrate data from rumen morphology, microbiome, metabolome, and epithelial transcriptome, thereby systematically analysing the differences in rumen function between barn feeding and traditional grazing conditions in Tibetan sheep. By elucidating the synergistic mechanisms among these multi-omics datasets, our study provides a novel perspective on the rumen adaptation of Tibetan sheep to different feeding regimes. This work also offers a robust theoretical foundation for the feeding management of plateau livestock.

## Methods

### Experimental design and sample collection

The experiment was conducted in the Haibei Demonstration Zone of Plateau Modern Ecological Husbandry Science and Technology (36°55′N, 100°57′E, altitude at 3,150 m) in Qinghai Province, China. A total of twelve one-year-old healthy castrated Tibetan sheep, all born as singletons with similar genetic backgrounds and initial body weights (BW: 31.75 ± 0.63 kg), were randomly assigned to two treatment groups (six sheep per group). The traditional grazing (TG) group was freely grazed on natural pasture comprising mainly *Kobresia humilis*, *Leymus secalinus*, *Elymus nutans*, and *Stipa purpurea* [22]. The barn feeding (BF) group was fed a TMR diet twice daily at 08:00 and 17:00 (diet composition is shown in Table S1). The experiment was performed from December to March and lasted for 105 d. Water was supplied ad libitum to the sheep during the experimental period. The details of the nutrient composition of diets were measured according to AOAC procedures and are provided in Table S2. At the end of the experiment, the Tibetan sheep were transported to a local abattoir for slaughter. After slaughter, 2 cm^2^ epithelial tissue samples of the dorsal rumen were collected and placed into a fixative solution for H&E staining. Approximately 5 g epithelial tissue samples of the dorsal rumen were rapidly collected, immediately frozen in liquid nitrogen, and subsequently stored at −80 °C for gene expression analysis [23]. Meanwhile, rumen fluid samples were collected and immediately frozen in liquid nitrogen for microbiome and metabolome analysis.

### Hematoxylin-eosin staining

Epithelial tissue samples of the dorsal rumen were fixed in 4% paraformaldehyde overnight. After dehydration in a series of gradient ethanol solutions (70%, 80%, 90%, and 95% absolute ethanol) and clearing using xylene, all samples were embedded in paraffin and sliced into 5 μm thick sections [24]. Sections were stained with H&E to examine the histological characteristics, and the morphological structures of papillae height, papillae width, and muscular thickness were determined using CaseViewer 2.2 (3DHISTECH Ltd., Hungary) under an electron microscope (Axio IMAGE Z2, Leica Microsystems Ltd., Germany) [25].

### 16S rRNA gene sequencing analysis

The DNA was extracted from the rumen fluid samples by using the TIANamp DNA extraction kit (TIANGEN, Beijing, China). The 16S rRNA gene targeting the V3–V4 region was amplified by PCR using composite-specific bacterial primers (338F 5′-ACTCCTACGGGAGGCAGCA-3′; 806R 5′-GGACTACHVGGGTWTCTAAT-3′). High-throughput sequencing was performed on the Illumina NovaSeq 6000 platform. After sequencing, the raw sequences were analyzed using USEARCH 10.0 [26]. The quality of the paired-end Illumina reads was checked using FastQC v.0.11.5 and processed using USEARCH. A feature table was generated using VSEARCH. The SILVA v123 database was used to classify the taxonomy of the representative sequences, and the plastids and non-bacteria were removed. For alpha diversity, Chao1 index was calculated. For beta diversity, variations in microbial composition between these two groups were investigated using principal coordinate analysis (PCoA). The LDA effect size (LEfSe) analysis was performed to identify differentially biomarkers across groups (LDA > 3).

### Metabolomics data analysis

Rumen fluid samples were centrifuged at 4 °C for 5 min at 10,000 r/min and transferred into a 1.5-mL tube, and pre-cold methanol with 10 μL internal standard 2-Chloro-L-phenylalanine were added. After centrifugation, 200 μL of supernatant was transferred to a fresh tube. Fifty microliters of each sample were removed and combined to prepare a quality control sample. After evaporation in a vacuum concentrator, 30 μL of methoxyamination hydrochloride was added and derivatized with 40 μL of BSTFA reagent at 70 °C for 1.5 h. All samples were then analyzed by GC–TOF-MS using an Agilent 7890 gas chromatograph system coupled with a Pegasus HT TOF/MS (LECO, St. Joseph, MI, USA). The resulting data were imported into SIMCA 14.1 software (Umetrics, Umea, Sweden) for orthogonal projections to latent structures-discriminant analysis (OPLS-DA). Differential metabolites were identified by combining the VIP values obtained from the OPLS-DA analysis and *t*-test (VIP > 1 and *P* < 0.05). Differential metabolites were identified and validated using the HMDB and KEGG. The data analysis tool MetaboAnalyst 5.0 was used to view the metabolic pathway distribution and enrichment of the differential metabolites.

### RNA isolation, sequencing, and bioinformatics analysis

Total RNA from the rumen epithelial tissue samples was extracted using TRIzol kit (Invitrogen, CA, USA). The quantity of the RNA was analyzed by NanoDrop 2000 spectrophotometer (IMPLEN, CA, USA), and the integrity was assessed by Agilent Bioanalyzer 2100 system (Agilent Technologies, CA, USA). Briefly, approximately 3 µg of total RNA from each sample was used to prepare an mRNA library according to the Illumina TruSeq™ RNA sample preparation protocol. Then, the library products were sequenced on an Illumina HiSeq 2500 sequencer. Clean data was obtained by removing the reads that contain sequencing adapter contaminations or poly-N and the low quality reads whose Q value were less than 20. At the same time, Q20, Q30, GC-content and sequence duplication level of the clean data were calculated and all of them were in good quality (with Q20 > 97% and Q30 > 92%). The sequences were aligned to the sheep reference genome (Oar_rambouillet_v1.0) using HISAT [27]. Sequence segments were spliced and annotated, and transcript expressions were calculated by RSEM. Fragments per kilobase of exon per million mapped reads (FPKM) was employed to quantify the gene expression. Based on negative binomial distribution, DEGs were screened out by using DESeq2 with *P* < 0.05 and |log_2_(fold change)| > 2. KEGG pathway enrichment analysis for the DEGs was performed by using KOBAS software. *P* < 0.05 was used to define KEGG pathways as significantly enriched.

### Statistical analysis

The statistical analyses on all other measures (except for the RNA sequencing, 16S rRNA sequencing, and GC–TOF-MS results) were analyzed using the *t*-test on IBM SPSS (version 22.0; SPSS Inc., Chicago, USA). The 13 DEGs, Top 20 different microbial genera, 19 different metabolites between the two groups were selected for Pearson correlation analysis. A *P* value of < 0.05 was considered statistically significant.

## Results

### Histological analysis of rumen epithelium

**Fig. 1:**
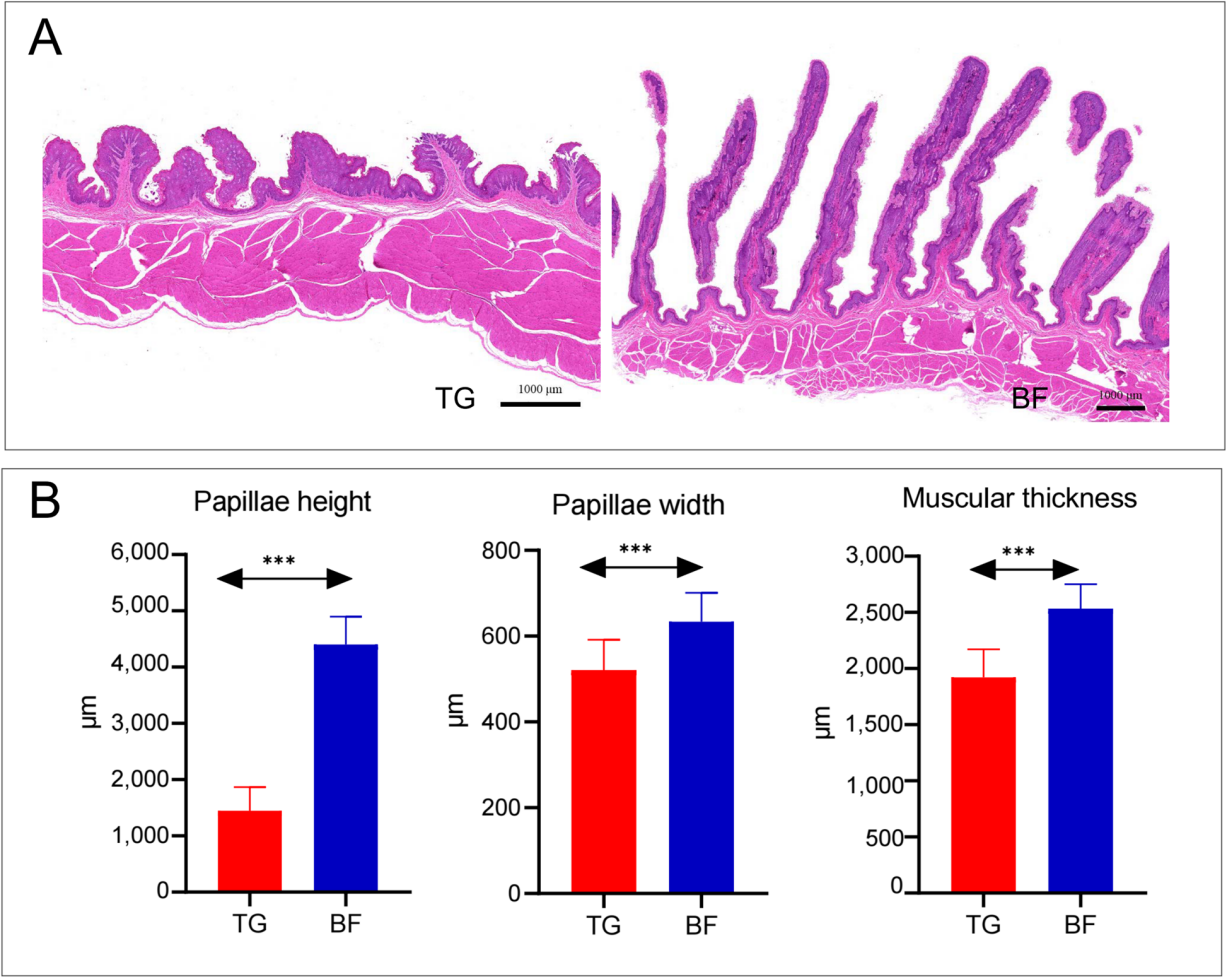
Morphological of rumen epithelial tissue of Tibetan sheep under different feeding regimes. **A** Rumen epithelium H&E staining. **B** Rumen papillae height, papillae width, and muscular thickness. ^***^P < 0.001

Figure 1 showed the morphological differences in the rumen epithelium development of two different groups. The papillae height and width of rumen epithelium and the muscular thickness of the rumen were all significantly increased in the BF group (*P* < 0.001).

### Rumen microbial profile and characteristics

**Fig. 2:**
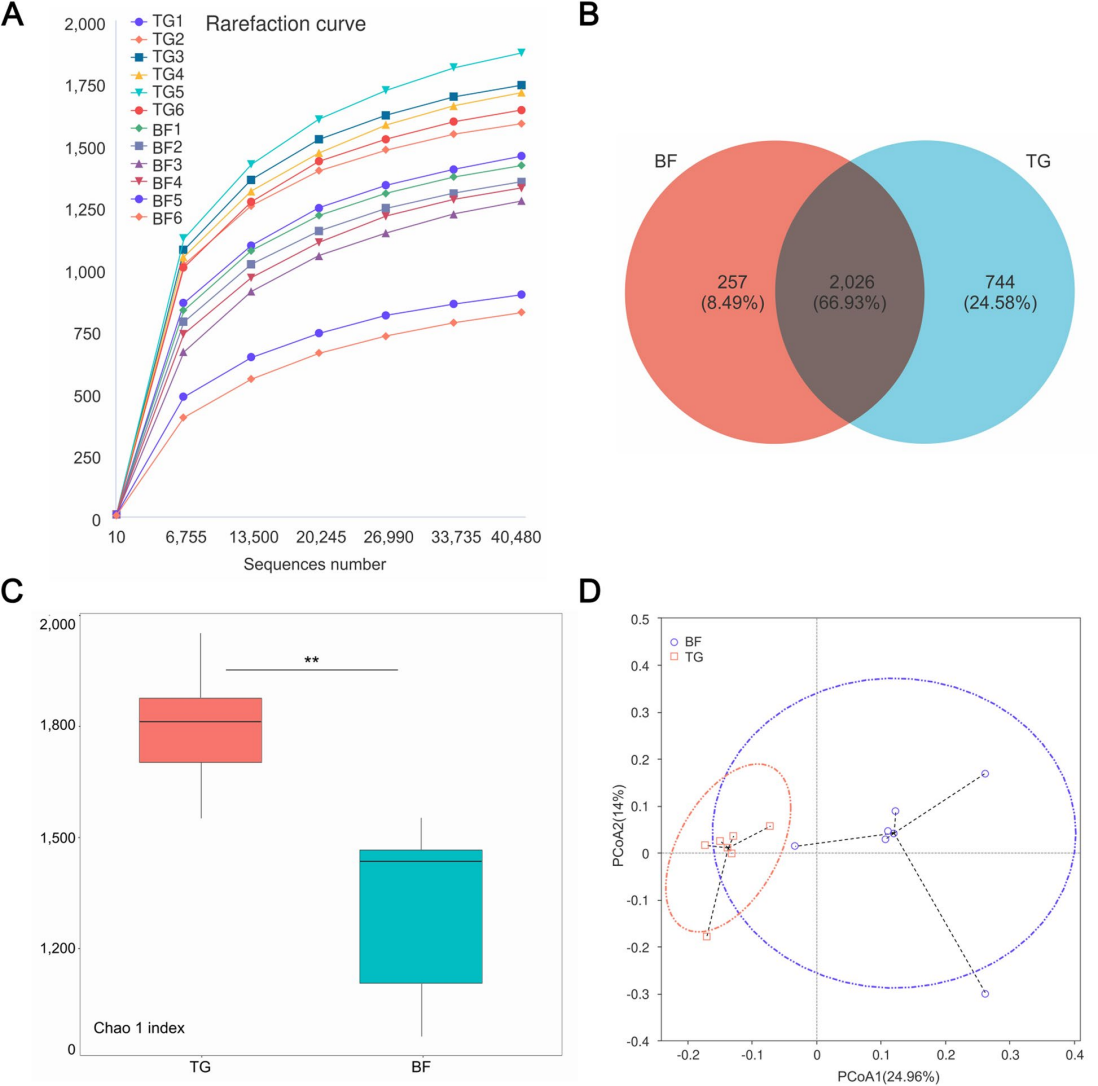
The characteristics in rumen bacteria of Tibetan sheep under different feeding regimes. **A** Rarefaction curve of samples. **B** OTUs venn diagram. **C** Chao1 index. **D** PCoA analysis. ^**^P < 0.01

The microbiota was analyzed in the two groups by sequencing the bacterial 16S rRNA V3 + V4 region. The rarefaction curves (Fig. 2A) of all samples obtained under the condition of 97% similarity reached a plateau, which indicated that the sequencing depth covered most of the microbiota in the samples. The Venn diagram showed that the groups shared 2,026 OTUs (66.93%), while the TG and BF groups had 744 (24.58%) and 257 (8.49%) exclusive OTUs, respectively (Fig. 2B). In addition, the Chao1 index was significantly higher in the TG group than that in BF group (*P* < 0.05; Fig. 2C). To address the effects of feeding regimes on beta diversity, unweighted UniFrac distance was used to characterize the bacterial community among samples (Fig. 2D). The PCoA result showed that the composition of the bacterial community of these two groups was largely separated from each other.

**Fig. 3:**
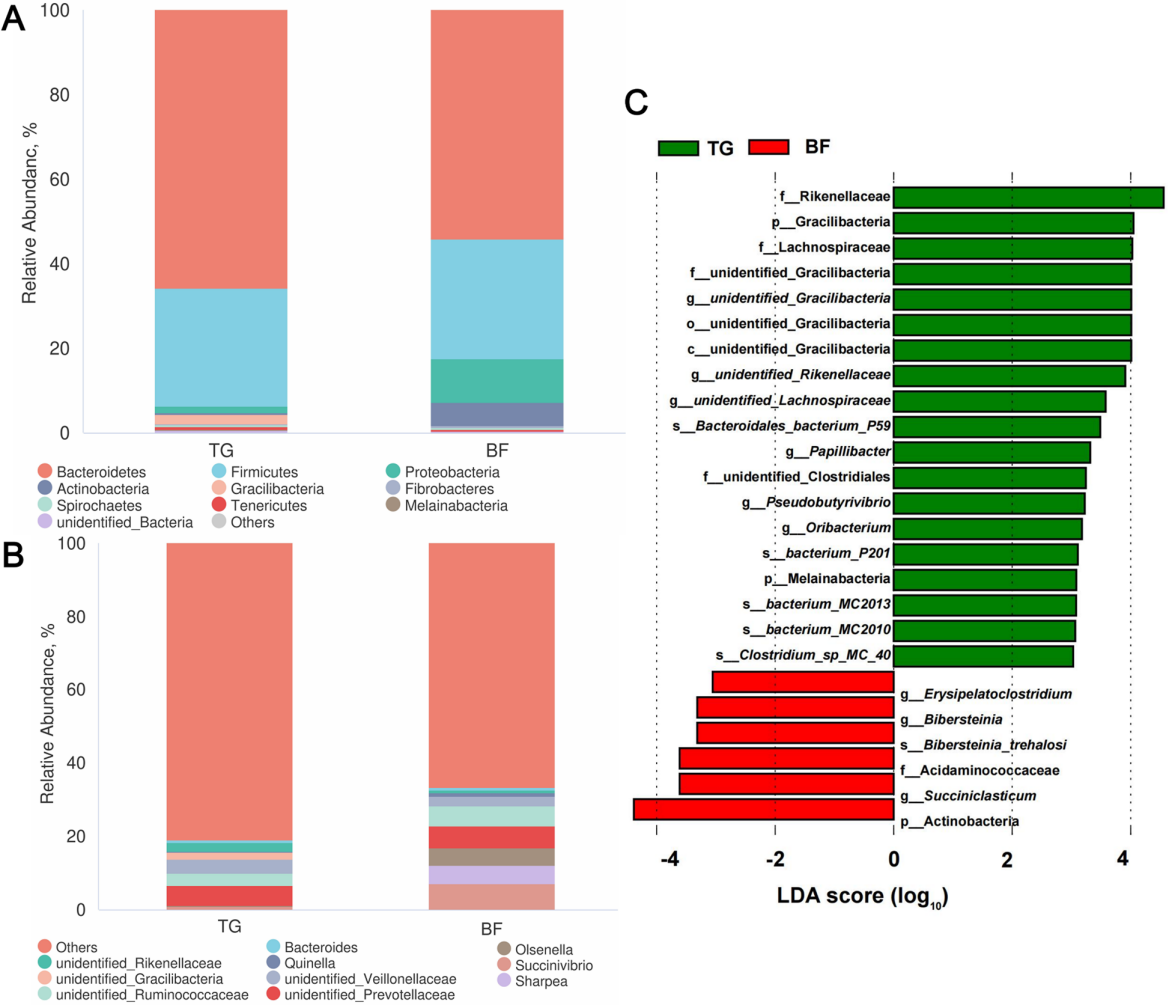
The changes in rumen bacteria of Tibetan sheep under different feeding regimes. **A** Composition of bacterial community at phylum level. **B** Composition of bacterial community at genus level. **C** LEfSe analysis with LDA scores of > 3 and *P* < 0.05

As shown in Fig. 3A, Bacteroidetes and Firmicutes were the predominant bacterial phyla in these two groups. They accounted for 65.7% and 27.8% in the TG group and 54.1% and 28.4% in the BF group, respectively. At the genus level (Fig. 3B), *unidentified_Prevotellaceae* and *unidentified_Veillonellaceae* were the most dominant genus in the TG group. However, the bacterial assemblages in the BF group were dominated by *Succinivibrio*, *unidentified_Prevotellaceae*, *Sharpea*, and *Olsenella*. As shown in Fig. 3C, LEfSe analysis revealed the difference in rumen microbiome between the two groups and the differences in the microbiome at various taxonomic levels (LDA > 3, *P* < 0.05). The results showed that 25 different bacterial taxa were found between these two groups. LEfSe showed that 19 bacterial taxa were significantly enriched in the TG group (e.g., Rikenellaceae, Gracilibacteria, and Lachnospiraceae), 6 bacterial taxa were enriched in the BF group (e.g., Actinobacteria, *Succiniclasticum*, and Acidaminococcaceae).

### Rumen metabolomic profiling based on GC–TOF-MS

**Fig. 4:**
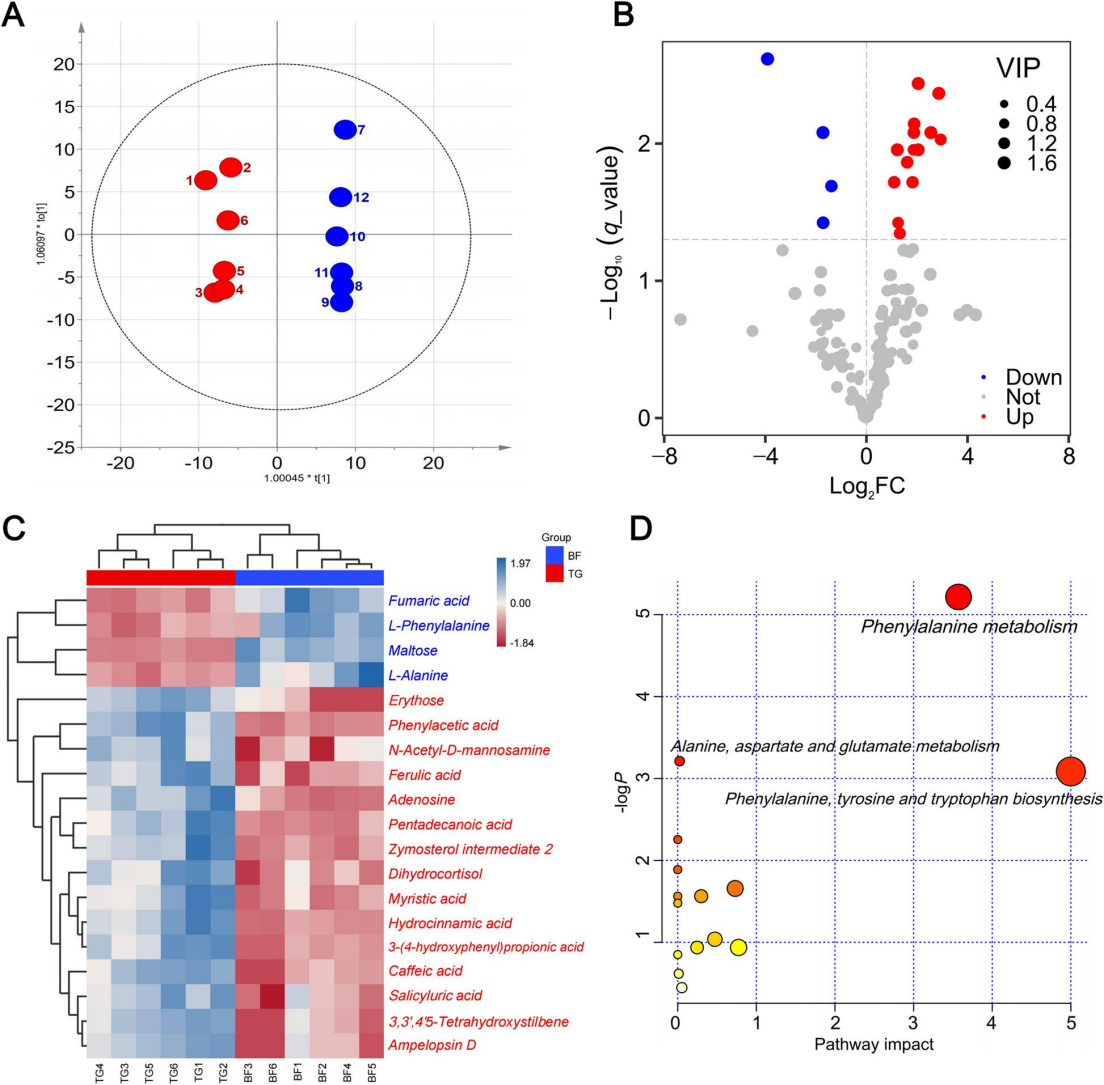
Rumen metabolomic profiling. **A** OPLS-DA scores. **B** Volcanic map and classification of differential metabolites. **C** Heatmap of significantly differential metabolites. **D** KEGG pathways based on the differential metabolites

After rigorous quality control and identification, 187 metabolites, including organic acids and derivatives, organoheterocyclic compounds, organic oxygen compounds, and benzenoids were obtained from the metabolomics library of these two groups. OPLS-DA was conducted to characterize the differences in rumen metabolic profiles between the different groups. The parameters for assessing the OPLS-DA model in different groups are represented in validation plots (Fig. 4A). The corresponding R^2^Y values of the OPLS-DA model for TG vs. BF was 0.811. This indicates that this model can be used to identify differences between the groups. Based on the statistical analysis results and the VIP values obtained from OPLS-DA, 19 metabolites (*P* < 0.05, and VIP > 1) were found to be significantly different (Fig. 4B and C, Table S3). Compared with the TG group, four metabolites (fumaric acid, maltose, L-phenylalanine, and L-alanine) in the BF group increased significantly (*P* < 0.05). Fifteen metabolites (e.g., phenylacetic acid, salicyluric acid, ferulic acid, and caffeic acid) were increased significantly in TG group (*P* < 0.05). Differential metabolites were analyzed using MetaboAnalyst 5.0 software to reveal their association with metabolic pathways (Fig. 4D). According to KEGG pathway identification, three pathways (phenylalanine metabolism, alanine, aspartate and glutamate metabolism, and phenylalanine, tyrosine and tryptophan biosynthesis) were significantly enriched (*P* < 0.05).

### Transcriptome profile analysis of the rumen epithelium

**Fig. 5:**
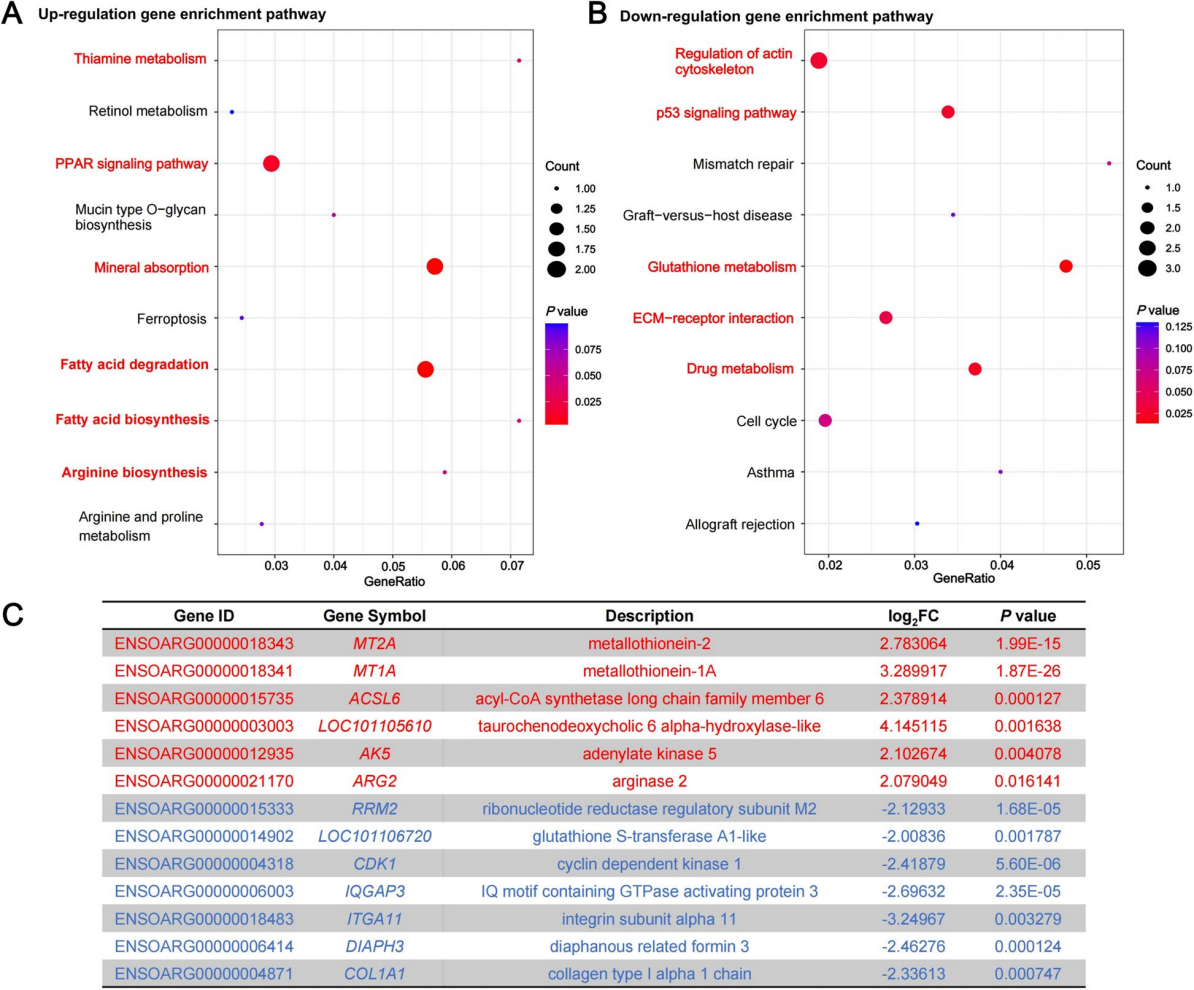
KEGG feature enrichment. **A** Up-regulation DEGs enrichment pathway. **B** Down-regulation DEGs enrichment pathway. **C** DEGs involved in above significantly KEGG pathways

A total of 210 DEGs were identified from the 19,383 genes in the rumen epithelial transcriptome data, including 100 up-regulated genes and 110 down-regulated genes form the TG vs. BF comparisons. Based on these identified DEGs, the KEGG functional enrichment analysis was performed. A total of six significantly changed KEGG pathways were identified for the 100 up-regulated genes in the BF group (Fig. 5A). Among them, KEGG enrichment results showed that the DEGs were mainly enriched in pathways related to nutrient metabolism, including fatty acid degradation, PPAR signaling pathway, fatty acid biosynthesis, mineral absorption, thiamine metabolism, and arginine biosynthesis. A total of five significantly changed KEGG pathways were identified for the 110 up-regulated genes in the TG group (Fig. 5B). Among them, KEGG enrichment results showed that the DEGs were mainly enriched in pathways related to glutathione metabolism, drug metabolism-other enzymes, p53 signaling pathway, regulation of actin cytoskeleton, and ECM-receptor interaction. Among these enrichment pathways, a total of six DEGs were up-regulated in the BF group (e.g., *MT2A*, *MT1A*, *ACSL6*, *LOC101105610*, *AK5*, and *ARG2*), and seven DEGs were down-regulated (e.g., *RRM2*, *LOC101106720*, *CDK1*, *IQGAP3*, *ITGA11*, *DLAPH3*, and *COL1A1*) (Fig. 5C).

### Rumen transcriptome-microbiome-metabolome joint analysis

**Fig. 6:**
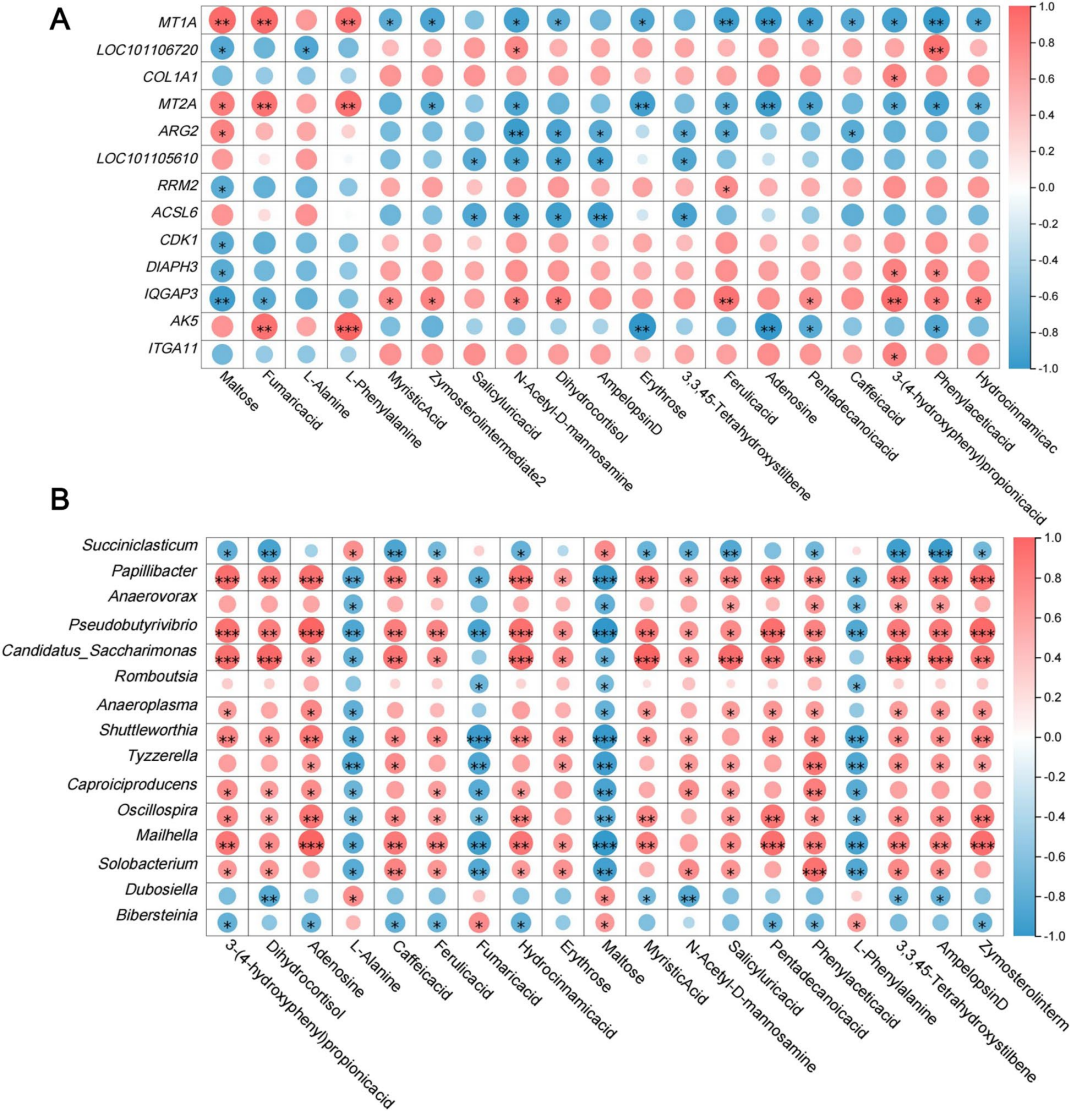
Rumen transcriptome-microbiome-metabolome joint analysis. **A** Interactions between DEGs and rumen metabolites. **B** Interactions between rumen microbies and rumen metabolites. ^*^P < 0.05, ^**^P < 0.01, ^***^P < 0.001

As shown in Fig. 6, correlation analyses between 13 DEGs, Top 20 different genera of bacteria, and 19 different metabolites were further performed. Maltose was significantly positively correlated with *MT2A*, *MT1A*, and *ARG2* (*P* < 0.05). Fumaric acid and L-phenylalanine were significantly positively correlated with *MT2A*, *MT1A*, and *AK5* (*P* < 0.05). L-Alanine was significantly negatively correlated with *LOC101106720* (*P* < 0.05). The findings of the correlation study between differential metabolites and differential microorganisms indicated that the four metabolites (maltose, fumaric acid, L-alanine, and L-phenylalanine) that were significantly up-regulated in BF exhibited a negative correlation with the majority of the microorganisms (12/20), including *Papillibacter*, *Pseudobutyrivibrio*, *Shuttleworthia*, *Oscillospira*, *Tyzzerella*, *Mailhella*, and *Caproiciproducens* (*P* < 0.05).

## Discussion

The rumen, a vital digestive and absorptive organ in ruminants, plays a pivotal role in the direct absorption and utilisation of degraded nutrients through the rumen epithelium. Consequently, the histomorphology of the rumen is closely related to the growth performance of ruminants. Morphological structures, such as rumen papilla height and papilla width, have been shown to determine the capacity of the rumen epithelium for nutrient uptake and ion transport [28]. The physical form of the feed, the ratio of concentrate to roughage, and the feeding method all play a key role in regulating the physiological and biochemical indexes of the rumen epithelium, such as epithelial morphology, pH, and the proportion of volatile fatty acids [29, 30]. The experiment demonstrated that there was an increase in papilla length and papilla width of the rumen epithelium in the BF group compared with the TG group. This finding suggests that the intake of high-nutrient-level rations is beneficial to the development of the rumen epithelium. Research has indicated that the inclusion of roughage in the diet has a substantial impact on the advancement of rumen papilla development, thereby suggesting that maintaining a suitable concentrate-to-roughage ratio is essential for the preservation of normal rumen papilla morphology [31]. As demonstrated by Shen et al. [32], the rumen papillae of goats exhibited greater development when fed high-energy and high-protein rations. In the present study, natural grass pasture exhibited lower levels of nutrients and poorer palatability, consequently resulting in lower levels of nutrient intake by Tibetan sheep and exerting a negative effect on rumen development.

The diversity and composition of rumen microbes are influenced by a multitude of factors, including animal species, dietary nutrition and the external environment [33, 34]. In this study, the Chao1 index of rumen microbes was found to be significantly higher in the traditional grazing Tibetan sheep than in the barn feeding group. This phenomenon may be closely related to the complexity of their natural grazing environment. The exposure of grazing Tibetan sheep to diverse vegetation types and soil microbial communities introduces a wider range of microbial sources to the rumen during foraging, thus contributing to microbial diversity [35]. Conversely, the homogeneous composition of the diet in the barn feeding group resulted in limited external microbial inputs, leading to homogenisation of the microbial flora. Consistent findings were observed in studies conducted on Tan sheep [36] and goat [37]. In terms of microbial community composition, the enrichment of Actinobacteria, *Succiniclasticum*, and Acidaminococcaceae in the rumen of barn feeding sheep is closely aligned with the metabolic demands of their high-protein and high-energy diet. Actinobacteria can degrade complex carbohydrates (e.g., starch and hemicellulose) and synthesize vitamin B_12_ [38, 39]. Their increased abundance likely enhances the efficient utilization of high-starch diets while supplying essential micronutrients to the host. *Succiniclasticum* obligately metabolizes succinate to propionate, which serves as the primary precursor for hepatic gluconeogenesis in ruminants [40]. The high abundance of *Succiniclasticum* indicates active glycolytic pathways in the rumen of barn feeding sheep, consistent with the energy supply pattern driven by high-carbohydrate intake. In contrast, the significantly enriched Rikenellaceae in the grazing sheep possess the ability to degrade complex plant polysaccharides [41], enabling the host to utilize high-fiber forage and compensate for the energy deficit of winter pastures. Additionally, Lachnospiraceae, as primary producers of butyrate, not only serve as an energy source for host epithelial cells but also exhibit anti-inflammatory properties and contribute to maintaining barrier integrity [42]. Their increased abundance may enhance ruminal mucosal immune tolerance, thereby mitigating challenges from pathogens or oxidative stress in grazing environments.

The significant elevation of metabolites such as fumaric acid, maltose, L-phenylalanine, and L-alanine in the rumen of barn feeding sheep reveals the profound impact of high-nutrient diets on rumen metabolism. Fumaric acid, a key intermediate in the tricarboxylic acid (TCA) cycle [43], indicating enhanced energy metabolism in rumen epithelial cells. This aligns with the superior rumen tissue development observed. Maltose, a product of starch degradation, reflects the high starch content of BF diets, suggesting stronger carbohydrate metabolic capacity. Notably, the marked increase in L-phenylalanine and L-alanine carries significant physiological implications. L-Phenylalanine serves not only as a critical substrate for protein synthesis but also as a precursor for bioactive compounds (e.g., tyrosine and dopamine) [44]. L-Alanine participates in gluconeogenesis through the glucose-alanine cycle, playing a pivotal role in energy metabolism [45]. Collectively, these differential metabolite profiles demonstrate that barn feeding conditions promote energy metabolism and protein anabolism in the rumen. Metabolic pathway enrichment analysis highlights the significant activation of phenylalanine metabolism and alanine, aspartate, and glutamate metabolism in barn feeding Tibetan sheep. The enrichment of these pathways reflects characteristic nitrogen metabolism traits, with heightened activity indicating enhanced amino acid metabolism and conversion capabilities [46]. The coordinated modulation of these metabolic pathways establishes the biochemical foundation for barn feeding Tibetan sheep to adapt to high-nutrient diets, providing a metabolic explanation for their superior rumen function.

In order to further explore the molecular level effects caused by cold season feeding strategies on the rumen of Tibetan sheep, the present study performed transcriptome sequencing of Tibetan sheep rumen tissues. In this experiment, a total of 210 DEGs were identified in two groups of Tibetan sheep under different feeding regimes. A comparison of the TG and BF groups revealed that the genes that were predominantly up-regulated in the BF group were predominantly enriched in nutrition metabolic processes, such as fatty acid degradation and PPAR signaling pathway. When ruminants are fed a high-nutrient diet, the content of short-chain fatty acids (SCFAs) in the rumen significantly increases [47, 48]. This metabolic change enhances fatty acid degradation, thereby promoting morphological development of rumen papillae (e.g., increased length and density) [49, 50], highlighting the driving role of nutrient supply in rumen development. Furthermore, *ACSL6* and *AK5* have been shown to synergistically promote lipid metabolism and energy production [51, 52], and elevated expression of these genes can promote the proliferation of rumen epithelial cells in barn feeding Tibetan sheep. Further analysis demonstrated that the carbohydrate- and fat-rich diet consumed by the barn feeding group specifically activated the PPAR signaling pathway. As a core regulatory network for lipid metabolism, this pathway facilitates fatty acid oxidation and lipid metabolism [53], providing sustained energy supply and supporting proliferation of rumen epithelial cells, ultimately forming a positive feedback loop between metabolic function and tissue development [54]. This mechanism aligns with the nutritional regulation theory of rumen development in ruminants, suggesting that stable high-energy diets under housed conditions are critical for optimizing rumen metabolic function. Compared to the barn feeding group, DEGs in traditional grazing Tibetan sheep were significantly enriched in the p53 signaling pathway and glutathione metabolism, indicating dual environmental pressures on the rumen microenvironment during cold-season grazing. First, composite stressors such as low temperatures, fluctuating forage quality, and exposure to soil-borne pathogens may induce DNA damage in rumen epithelial cells, subsequently triggering cell cycle arrest via the p53 pathway to maintain tissue homeostasis [55, 56]. Second, upregulation of glutathione metabolism reflects the host’s enhanced antioxidant defense system against free radical accumulation. As a central antioxidant, glutathione participates in diverse redox reactions, scavenges reactive oxygen species (ROS), and ensures normal physiological functions of proteins and enzymes by preserving their structural integrity [57]. Notably, the high cellulose content and low energy density of winter forage likely compelled grazing Tibetan sheep to adopt a "metabolic resource redistribution" strategy: limited energy intake prioritized immune defense over growth-related processes, which may explain the delayed rumen development observed in the grazing group.

Based on the above findings, we derive the following implications: For barn feeding Tibetan sheep during the cold season, the consumption of high-nutrient diets necessitates close monitoring of rumen metabolic balance to avoid subacute ruminal acidosis (SARA) risks, associated with excessive pursuit of economic benefits. We recommend adjusting dietary composition based on dynamic rumen pH monitoring. For traditional grazing Tibetan sheep, targeted supplementation of antioxidants or immunomodulatory substances could be implemented to alleviate oxidative stress caused by harsh environmental conditions, thereby promoting rumen health while maintaining environmental adaptability.

## Conclusion

**Fig. 7:**
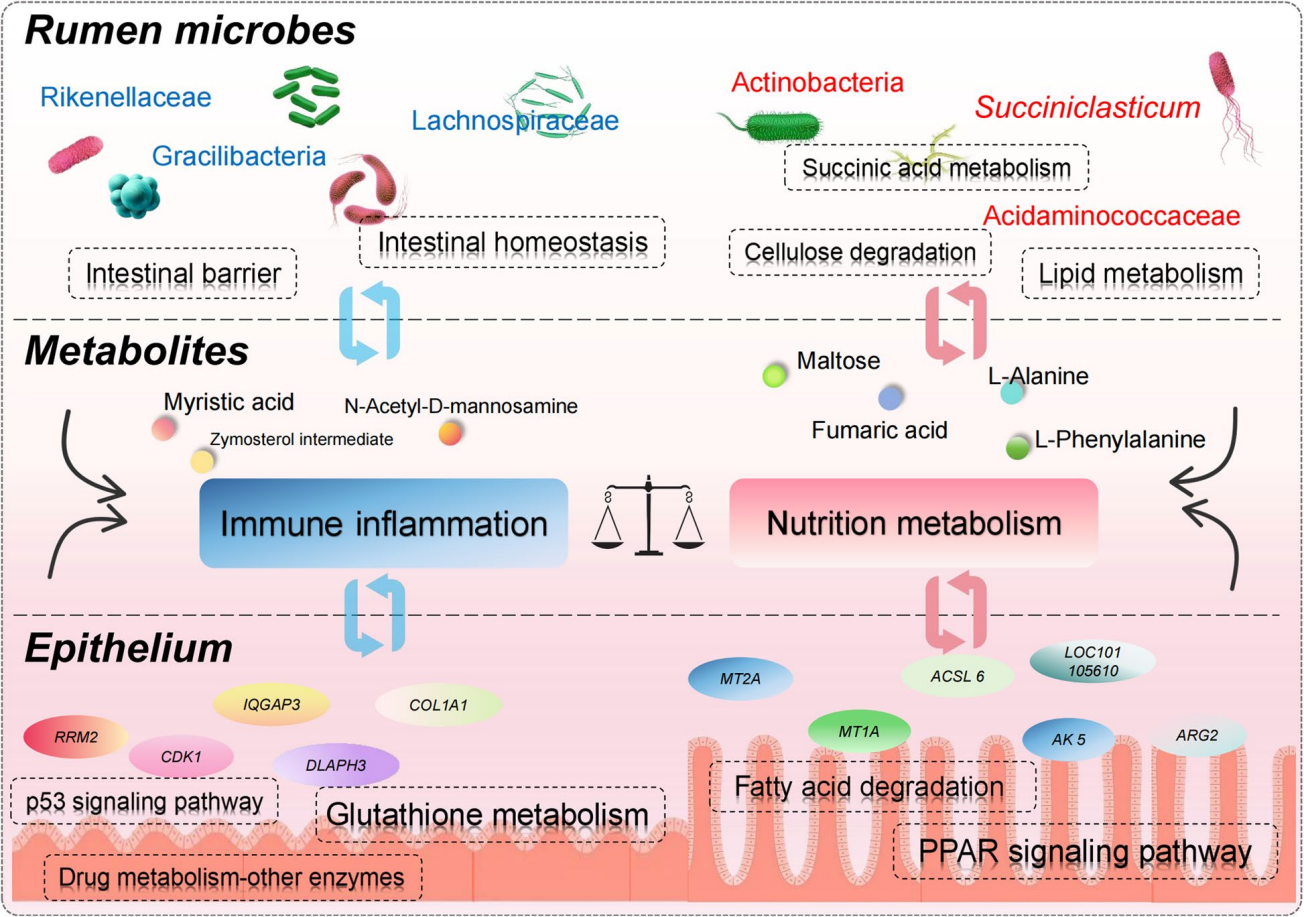
A schematic diagram summarising the response characteristics and multi-omics regulatory mechanisms of rumen function in Tibetan sheep under different feeding regimes in the cold season

This study systematically revealed adaptive differences in rumen function and their regulatory mechanisms between cold-season barn feeding and traditional grazing Tibetan sheep through integrated multi-omics analyses of rumen morphology, microbial communities, metabolites, and gene expression. Under cold-season barn feeding conditions, Tibetan sheep consumed high-nutrient diets, leading to the enrichment of carbohydrate-degrading microbiota (e.g., Actinobacteria and *Succiniclasticum*) in the rumen. These microbes generated metabolites such as fumaric acid, maltose, L-phenylalanine, and L-alanine, thereby activating lipid metabolism-related pathways (e.g., fatty acid degradation and PPAR signaling pathway) in rumen epithelium and promoting its development. In contrast, under traditional grazing conditions, exposure to low-quality forage and cold stress significantly increased the relative abundance of cellulose-degrading and antioxidant microbial communities (e.g., Rikenellaceae, Gracilibacteria, and Lachnospiraceae) in the rumen. This prioritized the activation of immune-related signaling pathways (e.g., p53 signaling pathway and glutathione metabolism) in rumen epithelium, favoring homeostasis maintenance at the expense of growth. Collectively, cold-season feeding strategies drive plastic adaptation of rumen function in Tibetan sheep by modulating microbial community structure, metabolic profiles, and host gene expression (Fig. 7). These findings provide multi-dimensional theoretical foundations for ruminant nutritional management and emphasize the importance of integrated environment-nutrition-microbe research.

## Supplementary Information

Additional file 1: Table S1 Diet composition of the TMR diet (on a dry matter basis). Table S2 Nutrient composition of the experimental diets (on a dry matter basis). Table S3 Identification of significantly different metabolites in rumen fluid between Tibetan sheep with different feeding regimes.

## Data Availability

The RNA sequencing data were deposited into the Genome Sequence Archive in National Genomics Data Center (Accession number: CRA024317). The raw reads of 16S rRNA gene amplicon sequencing were deposited into the NCBI Sequence Read Archive database (Accession Number: PRJNA1248067).

## Abbreviations

AOAC: Association of Official Analytical Chemists
BF: Barn feeding
DEGs: Differentially expressed genes
FPKM: Fragments per kilobase of exon model per million mapped fragments
HMDB: Human Metabolome Database
H&E staining: Hematoxylin-eosin staining
KEGG: Kyoto Encyclopedia of Genes and Genomes
LEfSe: Linear discriminant analysis effect size
OPLS-DA: Orthogonal partial least squares discriminant analysis
PCoA: Principal coordinates analysis
ROS: Reactive oxygen species
TG: Traditional grazing
