# Supplementary material for: Multi-omics analysis reveals host-microbe interactions driving divergent energy allocation strategies in Tibetan sheep under cold-season feeding regimes
Source: J Anim Sci Biotechnol. 2025 Sep 2;16:122. doi: 10.1186/s40104-025-01259-w (PMC12403966; doi:10.1186/s40104-025-01259-w)
Supplement: Supplementary file 1 — Additional file 1: Table S1 Diet composition of the TMR diet (on a dry matter basis). Table S2 Nutrient composition of the experimental diets (on a dry matter basis). Table S3 Identification of significantly different metabolites in rumen fluid between Tibetan sheep with different feeding regimes. [file 40104_2025_1259_MOESM1_ESM.docx]

**Table S1** Diet composition of the TMR diet (on a dry matter basis).

| **Ingredients, %** | **TMR diet** |
| --- | --- |
| Oat hay | 50.00 |
| Corn grain | 21.00 |
| Wheat grain | 13.50 |
| Wheat bran | 7.00 |
| Soybean meal | 3.50 |
| Rapeseed meal | 2.50 |
| NaCl | 0.50 |
| CaHPO_4_·2H_2_O | 0.30 |
| Bentonite | 0.50 |
| CaCo_3_ | 0.45 |
| NaHCO₃ | 0.25 |
| Premix ^1^ | 0.50 |

^1^ Premix provided per kg of feed: Vitamin A, 50,000 IU; Vitamin D_3_, 12,500 IU; Vitamin E, 1,000 IU; Cu, 250 mg; Fe, 12,000 mg; Zn, 1,000 mg; Mn, 1,000 mg and Se, 7.5 mg.

**Table S2** Nutrient composition of the experimental diets (on a dry matter basis).

| **Item, %** | **Group** | |
| --- | --- | --- |
|  | **TG** | **BF** |
| Crude protein, CP | 4.31 | 10.14 |
| Ether extract, EE | 1.05 | 2.72 |
| Neutral detergent fiber, NDF | 54.87 | 37.47 |
| Acid detergent fiber, ADF | 33.53 | 19.14 |

**Table S3** Identification of significantly different metabolites in rumen fluid between Tibetan sheep with different feeding regimes.

| **Super class** | **Metabolite name** | **RT ^1^** | **Mass** | **Similarity** | **Treatments** | | **VIP ^2^** | ***P* value** | **FC^c^(TG/BF)** |
| --- | --- | --- | --- | --- | --- | --- | --- | --- | --- |
|  |  |  |  |  | **TG** | **BF** |  |  |  |
| Benzenoids | Phenylacetic acid | 11.0425,0 | 164 | 912.607 | 7.10E-01 | 9.75E-02 | 1.568 | 0.004 | 7.28 |
|  | Salicyluric acid | 18.5588,0 | 179 | 330.500 | 2.14E-02 | 8.99E-03 | 1.211 | 0.038 | 2.38 |
| Hydroxycinnamic acids and derivatives | Ferulic acid | 19.9908,0 | 338 | 543.125 | 2.69E-03 | 7.33E-04 | 1.489 | 0.008 | 3.67 |
|  | Caffeic acid | 20.3985,0 | 219 | 521.000 | 1.11E-02 | 2.69E-03 | 1.585 | 0.004 | 4.14 |
| Lipids and lipid-like molecules | Myristic acid | 17.6117,0 | 117 | 832.036 | 4.72E-02 | 2.21E-02 | 1.420 | 0.019 | 2.14 |
|  | Pentadecanoic acid | 18.6125,0 | 117 | 660.222 | 4.35E-02 | 1.05E-02 | 1.553 | 0.011 | 4.13 |
|  | Dihydrocortisol | 28.3379,0 | 215 | 399.760 | 2.84E-03 | 9.30E-04 | 1.458 | 0.014 | 3.06 |
|  | Zymosterol intermediate 2 | 29.2409,0 | 215 | 505.074 | 6.93E-03 | 2.97E-03 | 1.556 | 0.011 | 2.33 |
| Nucleosides, nucleotides, and analogues | Adenosine | 24.4862,0 | 236 | 866.571 | 2.23E-02 | 6.03E-03 | 1.489 | 0.007 | 3.70 |
| Organic acids and derivatives | Fumaric acid | 11.6751,0 | 245 | 732.286 | 1.04E-02 | 3.44E-02 | 1.592 | 0.008 | 0.30 |
|  | L-Phenylalanine | 14.2376,0 | 120 | 774.929 | 2.09E-02 | 5.47E-02 | 1.422 | 0.020 | 0.38 |
|  | L-Alanine | 8.21195,0 | 116 | 966.179 | 4.77E-01 | 1.57E+00 | 1.482 | 0.038 | 0.30 |
| Organic oxygen compounds | Erythrose | 12.7736,0 | 201 | 571.960 | 3.14E-03 | 8.86E-04 | 1.387 | 0.019 | 3.54 |
|  | N-Acetyl-D-mannosamine | 20.1091,0 | 174 | 399.429 | 1.01E-02 | 4.05E-03 | 1.274 | 0.045 | 2.50 |
|  | Maltose | 25.436,0 | 204 | 928.714 | 4.25E-01 | 6.41E+00 | 1.652 | 0.002 | 0.07 |
| Phenylpropanoids and polyketides | Hydrocinnamic acid | 12.626,0 | 104 | 922.786 | 8.94E+00 | 1.17E+00 | 1.257 | 0.009 | 7.66 |
|  | 3-(4-hydroxyphenyl)propionic acid | 16.673,0 | 179 | 754.037 | 2.13E-02 | 3.64E-03 | 1.582 | 0.008 | 5.86 |
|  | Ampelopsin D | 26.6845,0 | 179 | 312.000 | 2.83E-02 | 8.10E-03 | 1.111 | 0.019 | 3.50 |
|  | 3,3',4'5-Tetrahydroxystilbene | 26.2821,0 | 73 | 657.480 | 3.58E+00 | 9.87E-01 | 1.137 | 0.011 | 3.63 |

^1^ RT represents retention time

^2^ VIP represents variable importance projection
